# An efficient sharding consensus algorithm for consortium chains

**DOI:** 10.1038/s41598-022-27228-1

**Published:** 2023-01-02

**Authors:** Xiaoxiong Wu, Wangxi Jiang, Mingyang Song, Zhenhong Jia, Jiwei Qin

**Affiliations:** College of Information Science and Engineering, Xinjiang University, Ürümqi, 830046 China

**Keywords:** Computer science, Information technology, Software

## Abstract

The consensus algorithm is very critical in any blockchain system, because it directly affects the performance and security of the blockchain system. At present, the classic Practical Byzantine Fault Tolerance Algorithm (PBFT), which is mainly used in the consortium chain, will lead to system communication congestion and reduced throughput when the number of nodes increases, so the PBFT algorithm is not suitable for large-scale consortium chains. In response to the above problems, this paper proposes a new clustering-based sharding consensus algorithm (KBFT), which aims to ensure that the consortium chain takes into account decentralization, security and scalability. The KBFT algorithm first uses the K-prototype clustering algorithm to shard the nodes in the network according to mixed attributes, and second, disjoint transactions are used to reach consensus in parallel in different shards. Concurrently, the KBFT algorithm introduces a supervision mechanism and a node credit mechanism, which is used to supervise and score the behavior of the nodes and select the proxy nodes, which improves security. We discuss the choice of shard size with the help of the binomial probability distribution and analyze the probability that the system can successfully form a global block under different node failure probabilities. Finally, the proposed algorithm is evaluated through theoretical analysis and simulation experiments. Results show that the proposed algorithm achieves a marked improvement in scalability and throughput along with a marked reduction in communication complexity compared with the classic baseline algorithm PBFT in this field of study, which improves the operating efficiency of the system and simultaneously guarantees the security and robustness of the system.

## Introduction

The essence of the blockchain is a decentralized distributed database, so the blockchain also faces the dilemma of the “impossible triangle”, that is, it cannot take into account decentralization, security and scalability at the same time. Decentralization is the core idea of the blockchain, which means that the blockchain no longer relies on central processing nodes. The rights and obligations of each node are equal, and the data in the system is jointly maintained by the nodes of the entire network. Security refers to the complete replication of data on each node to ensure the integrity of the data, and the use of relevant principles of cryptography for data verification to ensure that the data is not tampered with. Scalability also refers to transaction processing performance, mainly reflected in the throughput in the network. An important solution to blockchain scalability at present is sharding technology.

Currently, blockchain can be divided into three types according to the level of openness: public blockchain, consortium chain and private chain. Therefore, the consensus algorithm can correspondingly be divided into the public blockchain consensus algorithm, the consortium chain consensus algorithm and the private chain consensus algorithm. As the core technology of the blockchain, the consensus algorithm plays a vital role in the blockchain, so it is very important to design a consensus algorithm that can ensure the decentralization, security and scalability of the system as much as possible. Public blockchain consensus algorithms are typically proof-based, such as Proof-of-Work (PoW)^1^ and Proof-of-Stake (PoS)^2^. As the first consensus algorithm in blockchain technology, POW requires each node to compete for the right of accounting by calculating a mathematical problem but is not widely used due to the unfairness caused by the concentration of computing power and the huge power resources consumed by the calculation. The POS algorithm selects accounting nodes according to the amount of equity held. Although POS solves the problem of power consumption by the PoW algorithm, POS weakens decentralization. However, decentralization is the most essential feature of the blockchain. Decentralization makes the system highly fault-tolerant, because a decentralized system will not cause the entire network to stop working due to a single node failure. At the same time, the decentralized system is not easy to be attacked, because the attack of one or some nodes will not affect the operation of the entire system. Although the proof-based consensus mechanism has good node scalability, it produces problems such as low throughput and time delays. At the same time, the proof-based consensus mechanism will inevitably consume computing power (or memory resources), and when the scale increases, the waste of resources is more serious.

With the rapid development of blockchain technology, the current blockchain has developed from the era of public blockchain to the era of consortium chain, and consortium chain has become the preferred blockchain for many fields and applications. Concurrently, the consortium chain uses more light consensus algorithms, such as Paxos^3^, Raft^4^ and the classical Byzantine fault-tolerant algorithm PBFT^5^, among which Paxos and Raft are widely used in systems without Byzantine nodes. However, due to the variability and uncertainty of network attacks, there may be Byzantine nodes in the network. At this time, the PBFT algorithm has more prominent advantages than the Paxos and Raft consensus algorithms. Concurrently, PBFT does not require many calculations and has thus been widely used in consortium chains. In other words, Paxos and Raft can only tolerate faulty nodes and cannot tolerate Byzantine nodes.

In order to be able to design a consensus algorithm based on the PBFT algorithm and applicable to large-scale consortium chains, many scholars have performed extensive studies to improve the PBFT algorithm. For example, the EPBFT^6^ consensus protocol uses a verifiable random function (VRF) to select consensus nodes, making this protocol applicable to dynamic networks. Although EPBFT claims that it can reach a consensus under different failure conditions, if the master node fails to reach a consensus, it will still cause the communication overhead to reach Ο(N^2^) (N is the number of nodes in the network). The DGBFT^7^ consensus protocol markedly reduces the communication complexity through node grouping. Although DGBFT uses confidence in the selection of node grouping and proxy nodes, the protocol ignores a possible situation, that is, if a node has too high credit, it will always act as a proxy node, which may cause the blockchain to tend to be centralized .The CDBFT^8^ consensus protocol stimulates the enthusiasm of nodes through a voting reward and punishment scheme and its corresponding credit evaluation scheme. However, there is a problem that cannot be ignored in the CDBFT consensus protocol, that is, the designed credit model is too complicated, which will greatly reduce the efficiency of consensus. Therefore, the CDBFT consensus protocol is not suitable for consortium chain scenarios that require high efficiency. Although these protocols have improved the PBFT algorithm from different perspectives, with an increasing network scale, communication complexity is high, or the credit mechanism used is too complex. Therefore, it is important to design a consensus algorithm that can be applied to large-scale consortium chains. This paper proposes the KBFT algorithm, a new consensus protocol that can be used for large-scale consortium chains. The novelty of this algorithm lies in the combination of the consensus algorithm and K-prototype clustering algorithm for the first time, which markedly reduces communication complexity, improves system throughput, and sets up an efficient and fast credit mechanism and supervision mechanism to ensure the security and reliability of the system. The contributions of this paper can be summarized as follows:

- We first apply the K-prototype clustering algorithm to node sharding in the consortium chain. At the end of the cycle set by the system, KBFT will use the K-prototype clustering algorithm to reshape the nodes in the network to prevent nodes from jointly doing evil etc., thus ensuring the security of the system. Concurrently, during resharding, new nodes can choose to join the network. The addition of new nodes can expand the scale of the network and reduce the proportion of Byzantine nodes in the system.
- We use a consensus algorithm that combines the BLS^9^ multisignature and the Byzantine fault-tolerant algorithm to independently and in parallel achieve consensus on transactions in each shard so that the communication complexity in the network is markedly reduced and throughput increases linearly with the number of shards.
- We set up a simple and efficient credit mechanism and supervision mechanism for the algorithm to score and supervise the behavior of nodes, thus further ensuring the security of the system.

## Related work

Practical Byzantine Fault Tolerance (PBFT) is an improved consensus protocol based on the original Byzantine Fault Tolerance algorithm BFT. PBFT solves the problem of low efficiency of the BFT algorithm, and the complexity of communication changes from exponential to polynomial. However, there are still many problems with PBFT. For example, when the number of nodes is too large, communication between nodes will lead to high network communication overhead and network blocking. Generally, the number of nodes in the network will not exceed 100^10^. When the master node fails, the replica nodes will initiate a complex view change protocol to ensure the system's activity; thus, the efficiency of the system will be markedly reduced. PBFT does not have a punishment mechanism for malicious nodes, which will make malicious nodes stay in the network and continue to perform evil behavior. Based on the defects of the PBFT algorithm, many scholars have performed extensive research. Li et al.^11^ propose a scalable multilayer consensus mechanism based on PBFT, which divides nodes into different layers. Although the communication complexity is markedly reduced, the increase in the number of layers will still lead to longer transaction confirmation times. The GH^12^ divides the nodes in the network into groups, each with a primary node. The GH first achieves a local consensus among the nodes in the group, then the primary node achieves a global consensus. In literature^13^, the nodes are grouped by the consistent hash algorithm, and the communication complexity is markedly reduced under the design of this algorithm. Although the consensus algorithm proposed in the literature^11–13^ has marked advantages in scalability and communication complexity compared with the classical PBFT algorithm, it still produces considerable communication overhead when the scale of the blockchain network is large. Many scholars have started to solve these problems by simplifying the protocol. Zyzzyva^14^ uses speculative techniques to reduce the communication overhead cost and simplify the protocol so that the communication overhead is reduced to a value near its theoretical minimum. The fast Byzantine consensus^15^ generally requires only two communication steps for each request to reach a consensus. The HotStuff^16^ consensus algorithm uses the threshold signature algorithm, which makes the communication complexity linearly related to the number of nodes and reduces the number of signatures in the consensus process using the threshold signature. With the deepening of the research of consensus algorithms, the establishment of credit mechanisms and supervision mechanisms also plays a certain role in ensuring the security and reliability of the system. Based on the traditional PBFT algorithm, the protocol proposed in literature^17^ adds a trust value to the nodes and divides the nodes into different roles so that the system can reduce the communication overhead and improve the system security concurrently. The CDBFT^8^ defines a voting system based on credit rewards and punishments to markedly improve the enthusiasm of participants, eliminate malicious nodes, and improve the security and efficiency of the system. The RBFT^18^ algorithm combines the reputation model to evaluate the work of each node. Concurrently, nodes with higher reputation are more likely to become master nodes and package blocks, thus reducing the risk of master nodes being malicious nodes. Liu et al.^19^ propose a monitoring Byzantine fault tolerance mechanism, which adds a type of monitoring node to the network model. Monitoring nodes measure node behavior and determine the status of replicas in the blockchain. Monitoring nodes can guide new nodes to join the blockchain and use the trust scale to sense the status of nodes and eliminate malicious nodes or crashed nodes.

To increase the throughput of the system, the blockchain adopts new technologies such as sharding. Sharding technology was first used for database partitioning, and sharding technology divides large databases into smaller, faster, and more manageable parts. Applying sharding technology to blockchain, in terms of sharding strategy, sharding technology can be divided into three types: network sharding, transaction sharding and state sharding. The Elastico^20^ protocol is the first protocol that uses sharding technology in the blockchain consensus algorithm. However, because this protocol is designed for public blockchain, it requires economic incentives to encourage nodes to verify and is thus not suitable for consortium chain. Chen et al^21^. Proposed a new sharding consensus mechanism, which innovatively uses a combination of signature-based Anchorshash and jump consistent hash functions to construct shards, which improves the sharding rationality and security, and minimize remapping caused by shard changes. However, this algorithm has a serious problem that the process of node sharding is too complicated. If it is used in a real blockchain system, it will seriously affect the overall efficiency of the system.

Unsupervised learning is a popular machine learning technology that has been widely used in many fields. For example, abnormal data are found in massive big data, and many advertising platforms use this technology to subdivide users and various recommendation systems. The most commonly used scenarios for unsupervised learning are clustering and dimensionality reduction. Common clustering algorithms include K-means, DBSCAN, hierarchical clustering, etc., but these algorithms can only consider numerical data, while K-modes can only consider categorical attribute data. However, most of the data in engineering problems include both numerical data and categorical data; thus, a clustering method that can process two different types of data concurrently is required, and K-prototypes is such a method. In this article, we use the K-prototype clustering algorithm to sort nodes according to their mixed properties in the network. The proposed algorithm differs from those in the literature as follows:

- The KBFT algorithm can perform sharding quickly and efficiently through the K-prototype clustering algorithm.
- Compared with existing algorithms, the KBFT algorithm designs a simpler and more efficient credit mechanism and supervision mechanism based on sharding, making the network more secure and reliable.
- We analyze in detail the impact of shard size selection on security.

## System model of the algorithm

### System model

**Figure 1:**
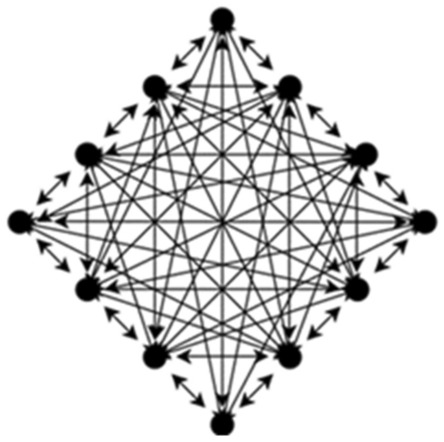
Single mesh network topology.

**Figure 2:**
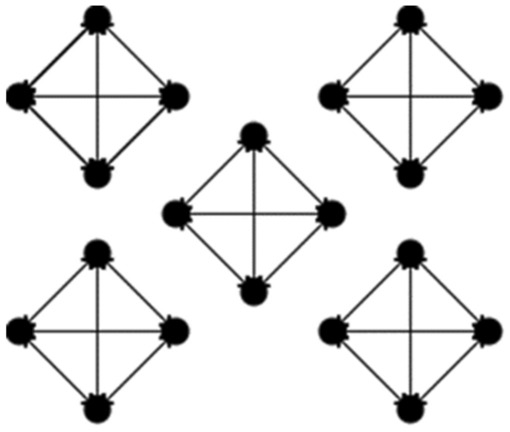
Multiple mesh network topology.

We improved a single mesh network topology into multiple mesh network topologies, the structure of which is shown in Figs. 1 and 2, and the model is formalized as follows. The target system is composed of the client, N consensus nodes and a supervisory node N_supervise_, and the N nodes are divided into different shards by the K-prototype clustering algorithm according to their numerical and classification attributes, which are represented by S. We let S = {S_1_, S_2_, …,S_m_} denote the division into m shards, where S_m_ is the mth shard. We also let T = {K_1_,K_2_, …,K_j_} denote the number of nodes in each shard; thus, N = K_1_ + K_2_ + $\cdots$  + K_j_. Selecting a node in each shard as a proxy node plays a role similar to that of the master node in the PBFT algorithm, but the algorithm selects a proxy node for each shard, which can markedly reduce the load of a single master node and increase the computing power of the master node. This node is no longer a bottleneck that affects the performance of the blockchain. The proxy nodes in each shard form the proxy committee, denoted by A = {a_1_, a_2_, …, a_m_}.

The supervisory node N_supervise_ set by the KBFT algorithm is the node that performs supervision in the consensus process, such as whether the consensus node actively participates in the consensus, whether there is any malicious change of information behavior, etc. N_supervise_ will locally store a list of all consensus nodes in a network to manage the information of all nodes, including the node’s public key, ID, IP address, credit, etc. The consensus nodes in the network fully trust the information recorded by N_supervise_. In order to maintain the distributed characteristics of the network and the decentralized characteristics of the blockchain to the greatest extent, N_supervise_ will not participate in the consensus process in the KBFT consensus algorithm, but only serves as a storage node for information.

**Figure 3:**
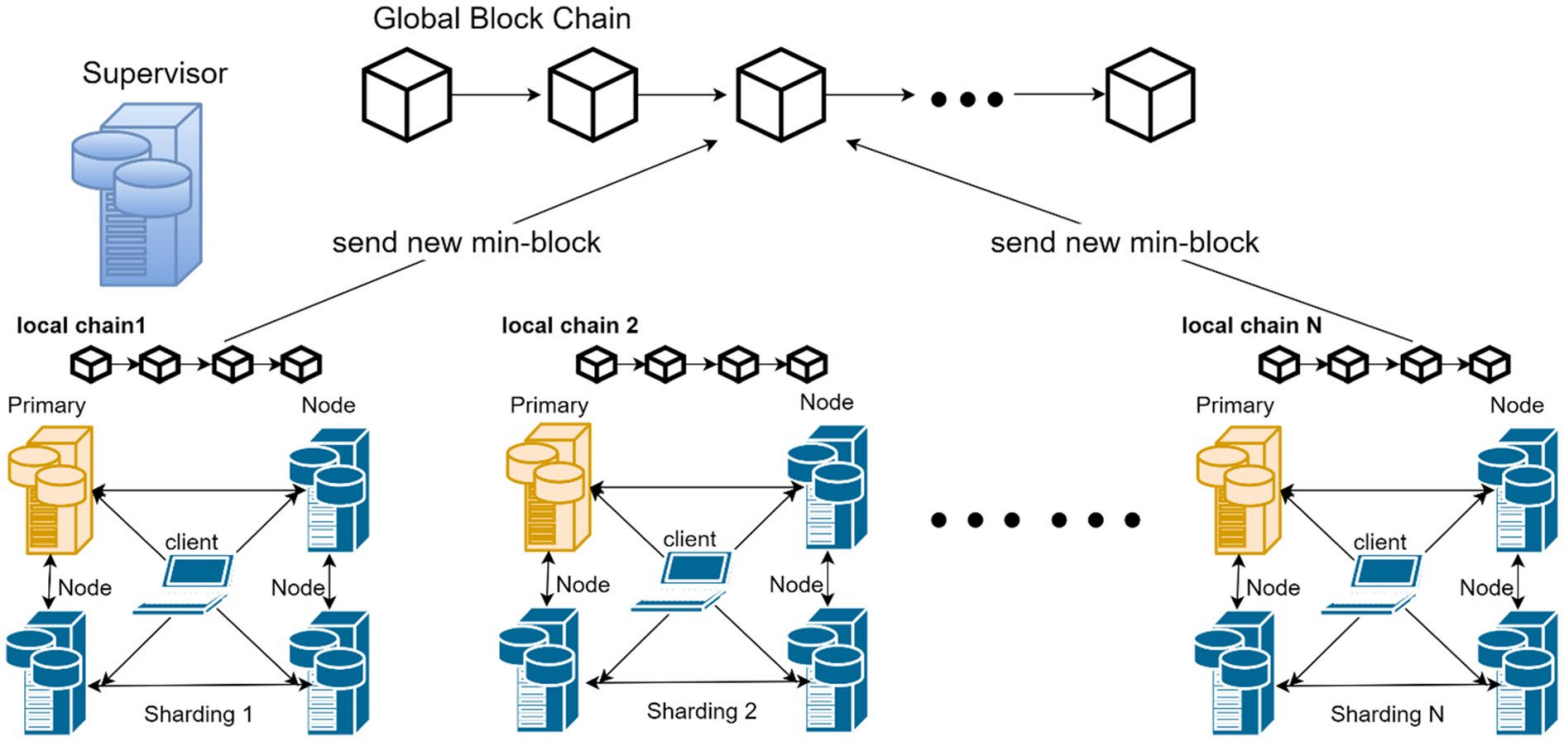
The overall system model.

The client that initiates the transaction signs the transaction and sends it to the proxy node of the shard. Inside the shard, consensus nodes verify this transaction through a Byzantine fault-tolerant algorithm based on the BLS multisignature. After consensus is reached, the block for the transaction will be stored locally on the node. However, the design of the KBFT algorithm does not allow each shard to retain only its own block data, but the global proxy node merges the blocks generated by each shard within the specified time and broadcasts it to the entire network so that the data stored by the nodes in the entire network are comprehensive. Because losing control of any shard will completely interrupt the blockchain, the comprehensiveness of data stored by nodes in the entire network is also in line with the original intention of decentralization and security of the blockchain. The overall system model is shown in Fig. 3.

### Security assumptions and threat models

- We divide the nodes in the network into different shards. The nodes in each shard are connected to each other through the network, and each node knows their basic information, such as the IP address and public key. Each shard is disjoint, and the nodes in different shards cannot communicate. We assume that the network connection between nodes in a shard is stable and that the network has weak synchronization characteristics. Once a node broadcasts any message, other nodes will arrive within the bounded time delay δ_t_.
- We assume that the adversary’s computing power is limited and cannot break the encryption technique or delay the network indefinitely; thus, we can use digital signatures or other encryption techniques to ensure the correctness of the message. The clients in this model are fault free, which can be guaranteed by client authentication.
- We stipulate that nodes joining the network must go through a strict identity access mechanism to prevent Sybil attacks.
- We argue that the proposed model is vulnerable to possible attacks that could interfere with the normal operation of the system. We assume that the probability of each node becoming a Byzantine node is $P_{F}$ and the number of Byzantine nodes in each shard is f and assume that the number of nodes in each shard is 3f + 1; thus, the number of Byzantine nodes that can be tolerated in each shard is one third of the number of nodes in that shard. The behavior of malicious Byzantine nodes may be arbitrary, such as refusing to participate in consensus, collaborating with other malicious nodes to attack the system or tampering with information. However, correct nodes will always follow the algorithm requirements.
- If the structure of shards in the network is fixed, malicious attackers may perform static loop attacks, slow adaptation attacks, etc., on nodes in the network. Therefore, the KBFT algorithm sets a constant time interval, and after the set time interval expires, the nodes in the network will be "reshuffled" to manage these attacks.

## KBFT algorithm design

### System flow of KBFT algorithm

**Figure 4:**
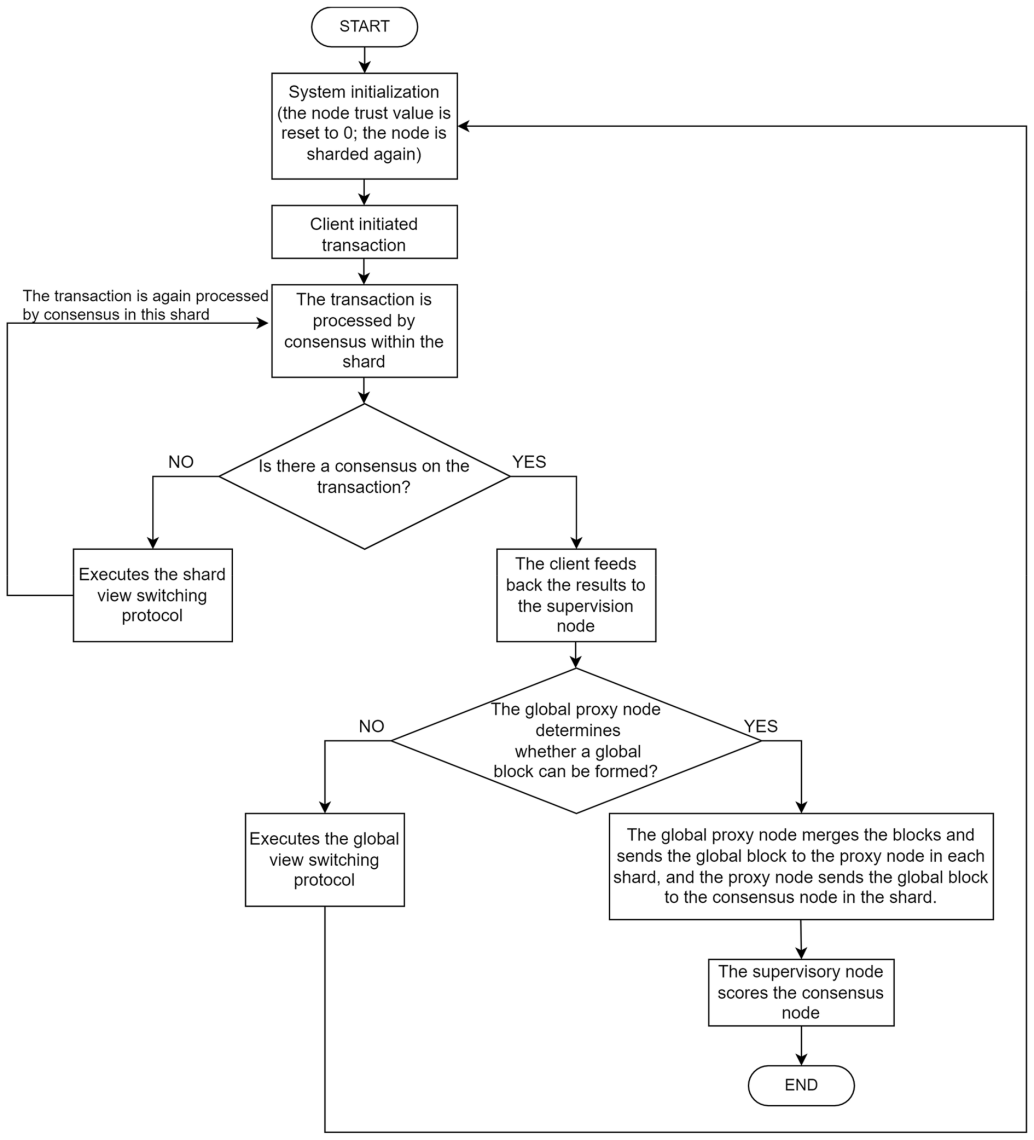
System flow chart of KBFT algorithm.

The KBFT algorithm first initializes the system, resets the trust value of the node to 0, and then uses the K-prototype clustering algorithm to segment the node. Secondly, the nodes in the shard perform consensus processing on the transaction initiated by the client. If the node reaches a consensus on the transaction, the client will feed back the consensus information to the supervisory node. If the consensus in the shard fails, the shard will start the view switching protocol in the shard to re-consensus process the transaction. Then, the shard proxy node sends the local block to the global proxy node. If the global proxy receives a legal number of blocks within the specified time, it will package all the blocks into a global block and send it to all proxy nodes. At the same time, the supervisory node will score all the nodes according to the information fed back by the client. If the global proxy node does not receive a legal number of blocks within the specified time, the system will start the global view switching protocol. The system flow of KBFT algorithm is shown in Fig. 4.

### Sharding of nodes

The KBFT algorithm first uses the K-prototype clustering algorithm to classify nodes according to their numerical attributes and categorical attributes. The numerical attributes include the node's ID, credit value, etc., while the categorical attributes include the company to which the node belongs, the node's IP address, and geographic location.

We let the node dataset be X = {X_1_,X_2_, X_3_, …, X_n_}, where n is the number of node objects in the dataset X, and each node data in the node dataset has m attributes (i.e., X_i_ = {X_i1_, X_i2_, X_i3_, …, X_ip_, X_i(p+1)_, X_i(p + 2)_, X_im_}, where there are p numerical attributes in the front and m-p categorical attributes in the back). Given a positive integer g, we divide the node dataset X into g shards. The sharding steps are as follows:

- We randomly select g nodes as the initial prototype (center point) and specify the initial prototype and the size of g according to the real application requirements in practical applications.
- The node objects are allocated to the nearest cluster according to the degree of dissimilarity, and the prototype of the cluster is updated after the allocation. The dissimilarity formula is as follows:
  1 $$d(x,y) = \sum\limits_{j = 1}^{p} {(x_{j} - y_{j} )^{{^{2} }} } + \gamma \sum\limits_{j = p + 1}^{m} {\delta (x_{j} ,y_{j} )}$$

where $\delta (x_{j} ,y_{j} ) = \left\{ {_{{1,(x_{j} \ne y_{j} )}}^{{0,(x_{j} = y_{j} )}} } \right.$ . The first term of Formula (1) is the Euclidean squared distance of the numerical attribute, and the second term is the simple matching dissimilarity on the categorical attribute.

- After the classification of the node is completed, the prototype of the category is redetermined, the mean value of the variable samples of the numerical type is considered to be the feature value of the new prototype, and the mode of the value of the variable samples of the categorical type is considered to be the new prototype feature value.
- We repeat steps (2) and (3) until no node samples change the category, and return the final sharding result.

### KBFT algorithm consistency protocol process

**Figure 5:**
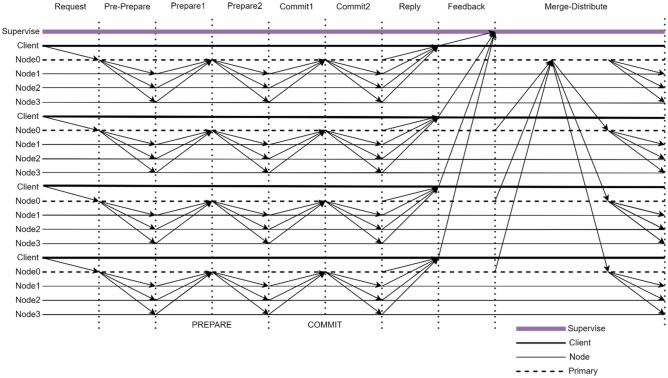
KBFT algorithm consensus protocol interaction process.

The KBFT algorithm includes a consensus process for each shard to process corresponding transactions and a process for merging and distributing block data. The consensus process occurs first, followed by the merge and distribution process. The basic flow of the algorithm is shown in Fig. 5.

The specific algorithm steps are as follows:

- *Request phase* The client sends the request message to the proxy node of the shard where it is located, and the consensus on the request will be performed within the shard.
- *Pre-prepare phase* A proxy node within a shard constructs a new block and broadcasts the block to the rest of the consensus nodes within the shard.
- *Prepare1 phase* The node verifies the block, and if the verification is valid, the BLS multisignature algorithm is used to sign, and the signature is fed back to the proxy node.
- *Prepare2 phase* The proxy node waits and collects valid signatures from other consensus nodes. After receiving at least 2f + 1 identically signed messages (including itself), the proxy node aggregates all the individual signatures into a BLS multisignature and then broadcasts this multisignature to all nodes. At this point, the PREPARE phase ends.
- *Commit1 phase* The node will verify that the received multisignature contains at least 2f + 1 signers and verify the transactions in the block broadcast by the proxy node during the pre-preparation phase. The node then signs the message received in the Prepare2 phase and sends it to the proxy node.
- *Commit2 phase* The proxy node waits and collects at least 2f + 1 valid signatures, aggregates these signatures together to form a BLS multisignature, and submits a new block with this aggregated signature. The new block is then broadcast to all nodes to verify the commit. At this point, the COMMIT phase ends.
- *Reply phase* When the node submits, the node sends a reply message to the client. When the client receives at least f + 1 identical confirmation messages from different nodes, the current request has reached the final consensus.
- *Feedback phase* When the client receives the reply messages in the shard, the client feeds back all the reply messages to the supervisory node. The supervisory node scores the behavior of all nodes according to the feedback results of the client. The supervisory node will analyze and compare the messages sent by the client. If it is found that the message sent by a certain node is different from that sent by other nodes, or if the message sent by a certain node is not received, it can be determined that the node has malicious behavior. Thereafter the supervisory node will score the nodes based on their behavior.
- *Merge-Distribute phase* The proxy nodes of each shard broadcast the formed local block to the global proxy nodes. The global proxy node merges all the blocks that it receives into a global block and distributes it to each shard proxy node. The proxy node that receives the global block will send the global block to all nodes in the shard. At this point, the consistency protocol process ends.

### Credit mechanism and supervision mechanism

The credit mechanism designed by the KBFT algorithm has three functions. First, the credit score adds an attribute to each node that can be used when sharding using the K-prototype clustering algorithm. Second, the credit value of each node will be changed after each consensus and will be kept by the supervisory node. When resharding, the system can quickly select the proxy nodes in the shard and the global proxy nodes according to the credit value. Finally, setting a credit mechanism can ensure that nodes work better and more honestly, thereby reducing the possibility of malicious nodes performing malicious acts. To improve the efficiency of the system, the credit mechanism designed by the KBFT algorithm is simple and efficient, and the node behavior can be quickly scored according to the node behavior without the need for a complicated score calculation process, and the supervisory node directly increases or decreases the credit value according to the behavior of the node. The credit value of honest nodes increases by 1 after each consensus, and the credit value of the nodes that do not participate in the consensus decreases by 1. The credit value of nodes with malicious behaviors will be reduced to 0 and will no longer participate in the relevant consensus before the global sharding. When a node has performed malicious acts multiple times, the node will be removed from the network and banned from joining the network. The calculation formula of the credit value is as follows.

2 $$N_{score} = \left\{ {\begin{array}{*{20}l} {N_{score} + 1} \hfill & {Honest\ node} \hfill \\ {N_{score} - 1} \hfill & {Not\ participating \ in\ consensus} \hfill \\ {0 } \hfill & {Show\ malicious\ behaviors} \hfill \\ \end{array} } \right.$$

### Dynamic resharding mechanism

The KBFT algorithm uses the mechanism of dynamic resharding to manage attacks against static sharding proposed in the threat model. After the time interval set by the network ends, the system uses the K-prototype clustering algorithm to reshape all nodes in the network. After each cycle, the number of nodes in the network and the numerical and categorical attributes contained in the same node are different from those contained before. This process ensures that after node sharding with the K-prototype clustering algorithm, the sharding result is different from the previous result, thereby preventing the system from being attacked by static sharding by resharding. At the same time, in order to comply with the original intention of blockchain decentralization, the trust value of each node will be set to 0 after each dynamic resharding. Because if the trust value remains unchanged, the node with a high trust value will continue to act as a sharding proxy node or a global proxy node after sharding, which does not conform to the characteristics of blockchain decentralization. At the same time, once the proxy node fails or is attacked, it will affect the security and efficiency of the network.

### Selection of shard proxy nodes and global proxy nodes

When the network is initialized, the system will randomly select or designate g nodes as the proxy nodes of each shard according to the real application. After the set period ends, the system will reshard all nodes in the network according to the information in the node information list stored by the supervisory node, and the selection of proxy nodes will no longer be random but will be based on the level of trust within the shard. Nodes with high trust degrees act as proxy nodes, which will make the system more secure and the consensus process more robust. The mechanism of selecting the global proxy nodes is similar to that of selecting the proxy nodes in the shard; thus, after selecting the proxy nodes in each shard, it will select the node with the highest credit in the selected proxy committee or select the global proxy node according to the real application.

### Joining of new nodes and exiting of nodes

While performing dynamic sharding, the number of nodes in the network can be increased or decreased. New nodes can join the network, or nodes can opt out of the network at this point. The addition of new nodes will improve the scalability of the network, and an increase in the number of nodes in the network will enhance the robustness of the network. Node exit includes the active exit of a node or its removal from the network as a penalty due to malicious behavior in a previous consensus. If malicious nodes always exist in the network without processing, then malicious nodes may gradually erode other good nodes, thus affecting the security of the network.

### View change

The normal operation of the KBFT algorithm is described above. However, some faults may occur during the operation of the network. For the faults in the partition, we primarily consider the behavior of the proxy nodes. Because the consensus in the partition is based on the PBFT algorithm, the algorithm can tolerate no more than one-third of the nodes doing evil concurrently. Therefore, it is more important to consider the behavior of the proxy nodes. The malfunction or mischief may be as follows:

- The proxy nodes in the shard did not broadcast the block information of the new transaction to other consensus nodes within the set time.
- After the consensus within the shard is completed, the proxy node does not send the block to the global proxy node, resulting in the global proxy node not receiving blocks with legal blocks within the set time.
- The global proxy node did not complete the work of receiving and merging and distributing the blocks of the entire network within the set time.

In response to these possible situations, the proposed algorithm sets up corresponding countermeasures to manage these situations reasonably to ensure the security and vitality of the system.

If a proxy node in a shard fails, the remaining correct nodes in the shard will choose a new proxy node by running a local view change, and then, the nodes continue to work towards reaching consensus. If the number of blocks received by the global proxy node within the specified time is less than the minimum threshold set by the network, then the system will perform an emergency resharding mechanism.

**Figure 6:**
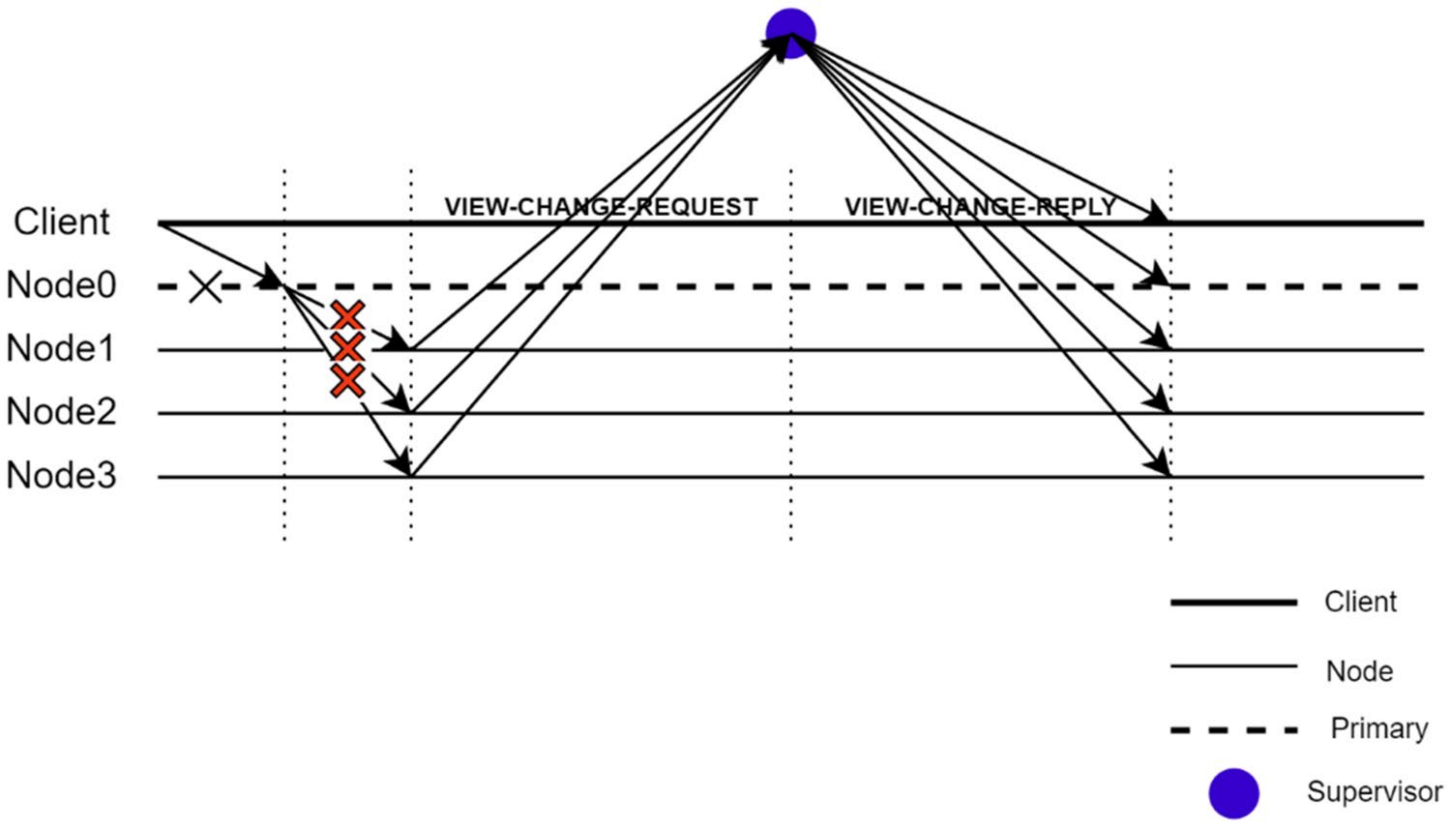
Intrashard view change flowchart.

View change within a shard: The view change protocol in KBFT is not as complex as that in PBFT. When the consensus in the shard is not completed within a specified time or the replica node does not receive the message sent by the proxy node, the shard will trigger the view change protocol in the shard. The specific process is that the replica node in the shard must only send a message requesting view change to N_supervise_. If N_supervise_ receives the same view change message sent by more than half of the nodes in the shard, N_supervise_ broadcasts the node with the highest trust value in the shard to the nodes and clients in the shard, and the shard consensus will restart. The flow chart of view switching within a shard is shown in Fig. 6.

Global view change: The global view change is also called the emergency resharding mechanism, which is different from resharding after the end of the cycle set by the network. The primary difference between the two is that the trigger timing is different. Emergency resharding is triggered when the number of blocks received by the global proxy node within the specified time is less than the minimum threshold set by the network or the global proxy node does not distribute the merged blocks. Both are performed by the K-prototype clustering algorithm. However, in the emergency resharding mechanism, if the global proxy node selected after resharding is still the same as the last time, the proxy node with the second highest trust value in the proxy node set will serve as the global proxy node.

## Security analysis

### Shard size selection

The size of the shard is important to the security of the system. The transaction generated by the client in each shard uses the Byzantine fault-tolerant consensus algorithm based on the BLS multisignature; thus, the number of Byzantine nodes that can tolerate each shard does not exceed one-third of the total number of nodes in the shard. We thus make the following two assumptions:、

- *Assumption (1)* The number of nodes in each shard is K (K = 3f. + 1, f = 1, 2, 3…).
- *Assumption (2)* The probability of failure of each node is $P_{F}$.

According to assumptions (1) and (2), the probability formula that each shard cannot successfully reach a consensus can be described by shown in Formula (3):

3 $$P_{Fail} = \mathop \sum \limits_{i = 0}^{{\left[ {\frac{2}{3}{*}K} \right]}} C_{K}^{i} P_{F}^{K - i} \left( {1 - P_{F} } \right)^{i}$$

**Figure 7:**
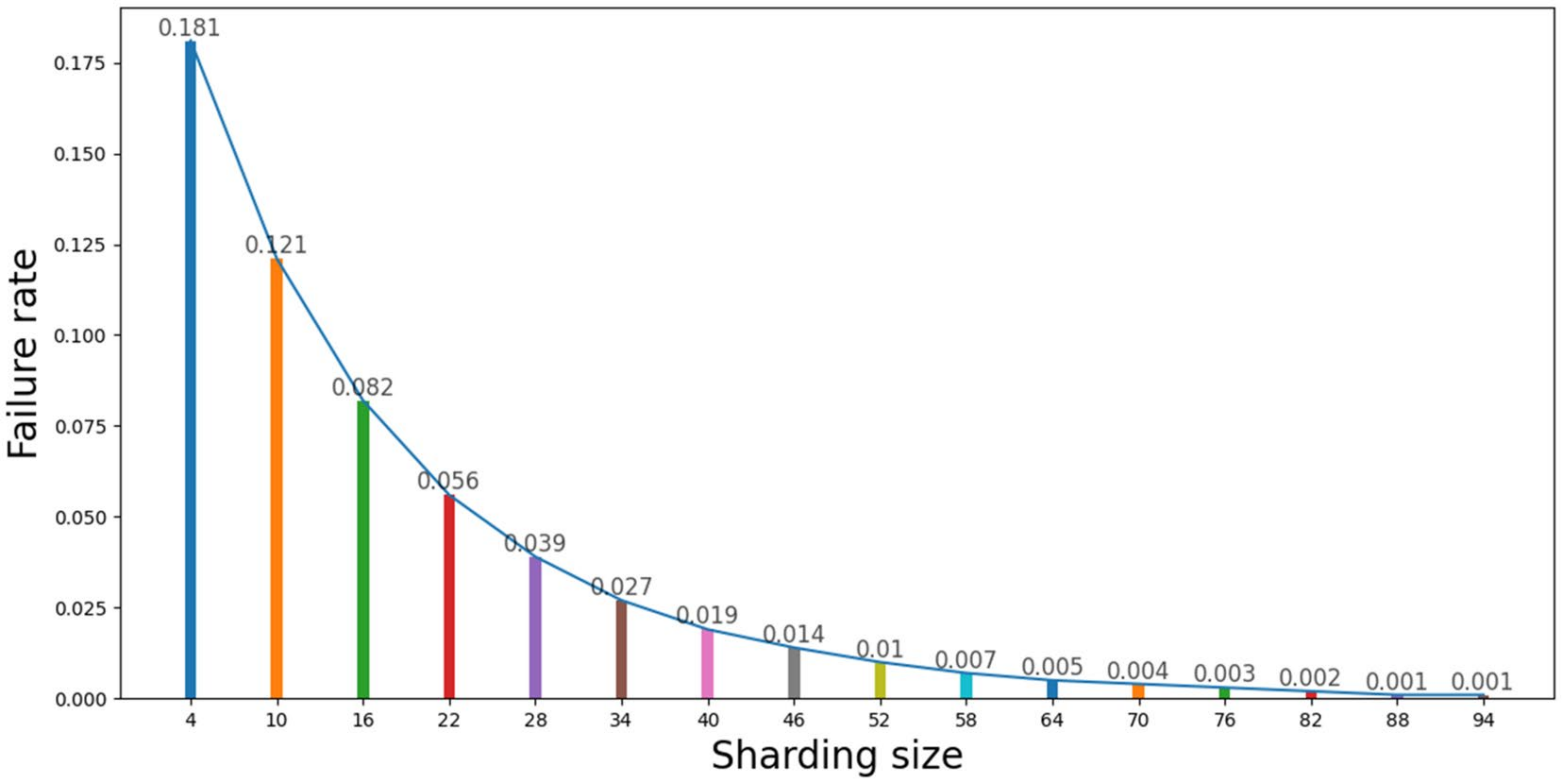
Intrashard consensus failure rate for different shard sizes.

We consider $P_{F}$ to equal 0.2 and visualize the function image of Formula (2), as shown in Fig. 7.

As shown in Fig. 7, when the probability of failure of each node is constant, and with a gradual increase in shard size, the consensus failure rate gradually decreases. When the number of nodes in the shard increases to 88, the probability of one-third of malicious nodes being included is one in a thousand; thus, the probability of consensus failure is negligible.

### Probability analysis of successful global block formation

In KBFT, we set a minimum merged block threshold, which is the minimum sum of blocks sent by the global proxy nodes from the proxy nodes in each shard. A complete consensus process is divided into two steps. The first step is the consensus within the shard, and the second step is the merging and distribution of blocks. Because the nodes in the network are divided into N/K shards, we must analyze the threshold of consensus required to keep the entire system secure and alive.

Event A: The faulty nodes in each shard do not exceed 1/3; thus, a consensus is successfully reached within the shard, and the probability of failure of each node is also $P_{F}$.

Event B: The number of blocks finally merged by the global agent node is greater than or equal to the set minimum threshold m; thus, the global block is successfully formed.

Therefore, we can derive the probabilities of events A and B from the binomial distribution as follows:

4 $${\text{P}}\left( {\text{A}} \right) = \mathop \sum \limits_{i = 0}^{{\left[ {\frac{1}{3}{*}K} \right]}} C_{K}^{i} (1 - P_{F} )^{K - i} (1 - P_{F} )^{i}$$

5 $${\text{P}}\left( {\text{B}} \right) = \mathop \sum \limits_{x = j}^{{\left[ {\frac{{\text{N}}}{K}} \right]}} C_{{\frac{{\text{N}}}{K}}}^{j} {\text{P}}\left( {\text{A}} \right)^{j} \left( {1 - {\text{P}}\left( {\text{A}} \right)} \right)^{{\frac{N}{K} - j}}$$

Substituting Formula (4) into Formula (5), the probability of success of the complete event B can be described by follows:

6 $${\text{P}}\left( {\text{B}} \right) = { }\mathop \sum \limits_{x = j}^{{\left[ {\frac{{\text{N}}}{K}} \right]}} C_{{\frac{{\text{N}}}{K}}}^{j} \left[ {\mathop \sum \limits_{i = 0}^{{\left[ {\frac{1}{3}{*}K} \right]}} C_{K}^{i} (1 - P_{F} )^{K - i} (1 - P_{F} )^{i} } \right]^{j} \left[ {1 - \mathop \sum \limits_{i = 0}^{{\left[ {\frac{1}{3}{*}K} \right]}} C_{K}^{i} (1 - P_{F} )^{K - i} (1 - P_{F} )^{i} } \right]^{{\frac{N}{K} - j}}$$

**Figure 8:**
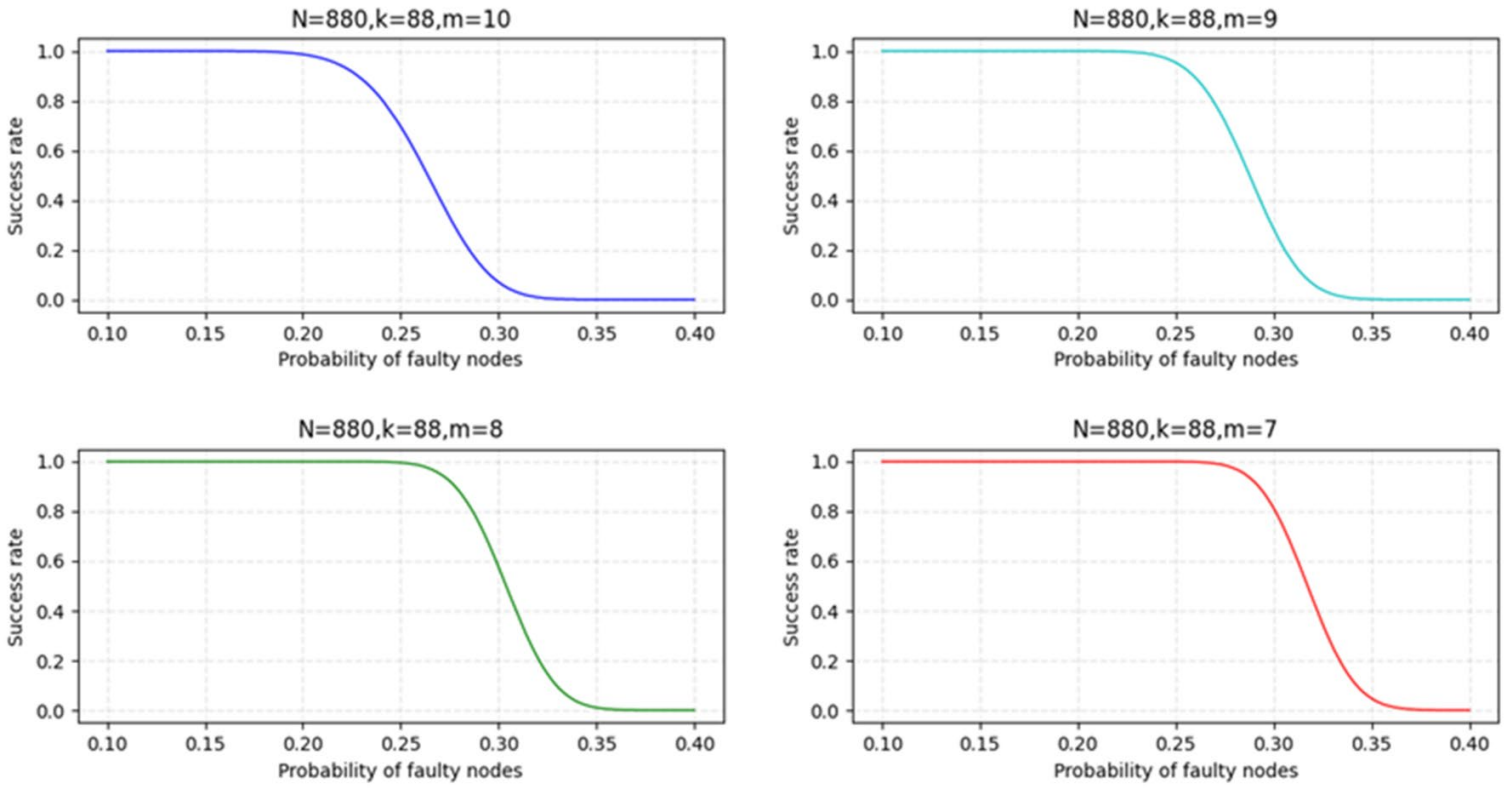
Analysis results of the relationship between success rate and $P_{F}$ under different m and the same K and N.

To more intuitively visualize the relationship between the success rate of successfully forming the global block and $P_{F}$, we visualize the function image of Formula (6) under different m and the same K and N, as shown in Fig. 8.

As shown in Fig. 8, when the number of nodes in the network equals the number of nodes in the shard, the inflection point in the probability curve is gradually delayed as the threshold of the minimum merged block decreases. Concurrently, if the probability of failure of each node is smaller, the success rate is higher. When the node failure probability can be controlled to be less than or equal to 0.2, the KBFT algorithm can guarantee a success rate of 100%.

## Analysis of KBFT algorithm

### Communication overhead

Assuming that the total number of nodes in the KBFT and PBFT algorithm networks is both N, and to avoid losing generality, we assume that the number of nodes in each shard in KBFT is K; then, the network is divided into N/K shards. According to the PBFT communication overhead calculated in literature^7^ and the consensus protocol interaction process of the KBFT algorithm in Fig. 4, two conclusions can be drawn as follows:

- *Conclusion 1* The communication complexity required by the PBFT algorithm to complete a consensus is Ο(N^2^), and the specific communication overhead is:
  7 $$C_{{{\text{PBFT}}}} = 2{\text{N}}^{2}$$
- *Conclusion 2* The KBFT algorithm performs node sharding, and each shard independently performs Byzantine fault-tolerant consensus based on the BLS multisignature. Therefore, the complexity of completing a consensus is Ο(Ν), and the specific communication overhead is:
  8 $$C_{{{\text{KBFT}}}} = 7{\text{N}} - \frac{{2{\text{N}}}}{{\text{K}}} - 2$$

We let the ratio of the communication overhead of the two algorithms be J, and Formula (9) can be obtained from Formula (7) and Formula (8) as follows:

9 $${\text{J}} = \frac{{C_{{{\text{KBFT}}}} }}{{C_{{{\text{PBFT}}}} }} = \frac{{7{\text{N}} - \frac{{2{\text{N}}}}{{\text{K}}} - 2}}{{2{\text{N}}^{2} }}$$

**Figure 9:**
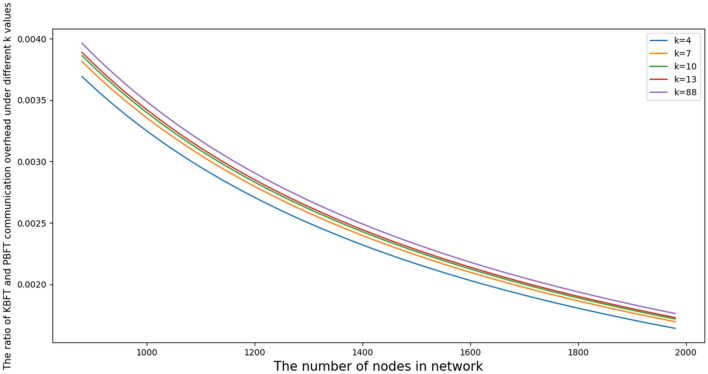
2D graph of the ratio of communications.

**Figure 10:**
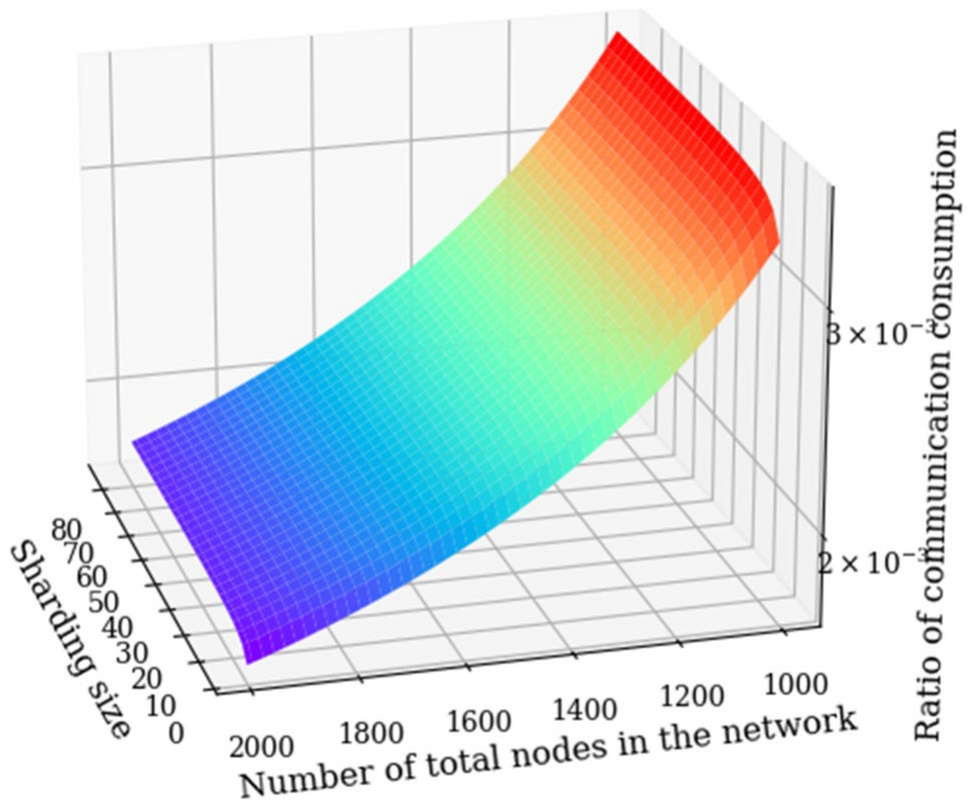
3D graph of the ratio of communications.

Figures 9 and 10 are 2D and 3D function diagrams of J, respectively, where K in Fig. 9 are 4, 7, 10, 13 and 88, respectively. The range of K in Fig. 10 is [4,88].

According to Fig. 9, when the number of nodes in the network is the same, their communication ratio decreases as the number of nodes in a fragment decreases, indicating that the smaller the number of nodes in a fragment is, the better the KBFT algorithm can reduce the communication overhead. Second, when the shard size is fixed, the ratio of the communication overhead of the two algorithms will gradually decrease as the number of network nodes increases, indicating that the larger the network scale is, the more advantageous the KBFT algorithm has in node communication resources.

As shown in Fig. 10, when the total number of nodes in the network using the PBFT algorithm and the KBFT algorithm are the same large number, even if K changes, the communication overhead ratio of the two is nearly constant. According to Formula (9), the denominator will be much larger than the numerator when N is large; thus, when N is fixed, the influence of the change of K on J will be nearly unchanged.

### Throughput

Throughput is defined as the total amount of transaction transactions that the network can process per unit time, which is expressed in Formula (10) as follows:

10 $$T{\text{PS}} = \frac{{T_{x} }}{{\Delta_{time} }}$$

where $T_{x}$ is the total transaction volume packaged into the block by $\Delta_{time}$, and $\Delta_{time}$ is the time interval from the completion of the transaction creation to the transaction on the chain. We assume that the total transaction volume generated in the system using the PBFT algorithm and the KBFT algorithm per unit time is the same. We assume that the total volume generated per unit time is the same in systems using PBFT and KBFT; that the number of nodes participating in the consensus is the same in the two systems; and that the volume contained in a block is the same. The former will process the transaction by all nodes to reach an agreement; thus, all nodes throughout the network have the same transaction consensus, while the latter will shard the transaction, and each shard processes different transactions. Therefore, the throughput of the system using the KBFT algorithm will be N/K times that of the system using the PBFT algorithm. N/K is the number of shards in the network, and because KBFT uses network sharding and transaction sharding, the total throughput increases linearly with the increase in the number of shards.

### Reliability and robustness

The proxy nodes in each shard of the KBFT algorithm and the global proxy nodes are selected according to the credit value. Nodes with high credit values can lead other consensus nodes in the shard to complete the consensus, to effectively promote the efficiency of system operation, and to ensure the safety and reliability of the entire system. Concurrently, the nodes are scored according to the consensus behavior, which can encourage honest nodes to perform better, while malicious nodes will reduce their credit value or even be eliminated from the network due to malicious behavior. If the master node is a Byzantine node, then complex switching views will be performed to achieve reconsensus, which will markedly reduce efficiency. If the master node is a Byzantine node, the consensus will fail, and the remaining replica nodes will execute a complex view switching protocol to select a new master node for reconsensus, which will markedly reduce efficiency. The PBFT algorithm also does not impose any penalty on the malicious behavior of Byzantine nodes, which may cause Byzantine nodes to continue malicious behavior without paying any price.

Concurrently, the KBFT algorithm is not designed for blockchain networks with a fixed number of nodes. New nodes can choose to join the network while the network is resharding. The increase in the number of nodes in the network reduces the proportion of malicious nodes, thus ensuring the robustness of the system and improving the security of the system.

### Simulate consensus

We use Python to conduct a simulation experiment of the KBFT algorithm. In the simulated consensus, we set the failure probability $P_{F}$ of a single node as the independent variable and the success rate P(B) as the dependent variable. Concurrently, the consensus simulation sets the total number of network nodes to 880, the number of nodes K in the shard to 88, and the minimum merged block threshold m to 7, 8, 9, and 10. The reason why the number K of nodes in each shard is set to 88 is because according to Fig. 7, when the number of nodes in the shard is 88, the failure rate of reaching consensus within the shard will be reduced to 1 in 1000.

**Figure 11:**
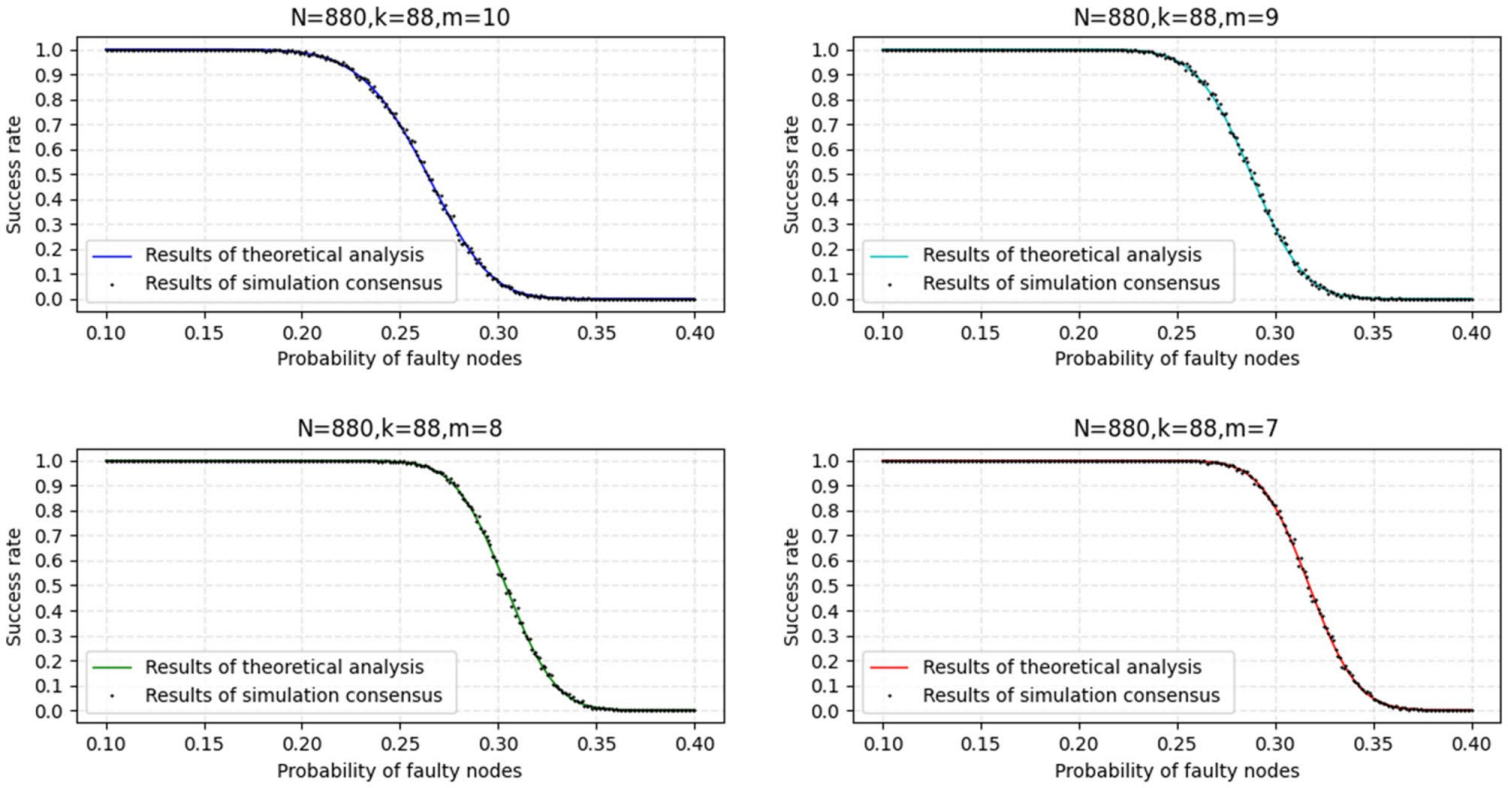
Simulated consensus results.

The results of the simulated consensus experiment repeated 1000 times are shown in Fig. 11 below.

Figure 11 shows that the curve obtained from the simulation consensus experiment repeated 1000 times accurately fits the curve obtained according to Formula (6) in the fifth part, which is sufficient to show that the proposed probability analysis for the successful formation of global blocks in the fifth part is correct. Therefore, the conclusion obtained from Fig. 8 is applicable to the analysis of Fig. 11; thus, if the probability of failure of each node is small, the KBFT algorithm can guarantee the success of 100%, which can be controlled for the blockchain that requires an identity access mechanism, such as the consortium chain. Concurrently, the credit and supervision mechanisms designed by the KBFT algorithm can also ensure that the probability of node failure is small.

The simulated consensus experiments conducted in this study are consistent with the assumptions made in the analysis of successful global block formation in the fifth part, which makes it meaningful to compare the two. By combining theoretical analysis with simulated consensus experiments, we can demonstrate that the proposed simulated consensus experiments are effective and accurate.

## Comparison of consensus algorithms

We list and compare several improved consensus algorithms based on PBFT algorithm in Table 1. The main comparison indicators are communication complexity, throughput, node sharding, credit mechanism, supervision mechanism, and Byzantine fault tolerance.

**Table 1:** Comparison of consensus algorithms.

	Communication complexity	TPS	Node sharding	Credit mechanism	Supervision mechanism	Byzantine fault tolerance
PBFT^5^	High	Low	No	No	No	Yes
Multi-Layer-PBFT^11^	High	Low	Yes	No	No	Yes
NBFT^13^	High	Low	Yes	No	No	Yes
GH^12^	High	Low	Yes	No	No	Yes
CDBFT^8^	High	Low	No	Yes	No	Yes
RBFT^18^	High	Low	No	No	Yes	Yes
Algorithm^21^	High	Low	Yes	No	No	Yes
KBFT(proposed)	Low	High	Yes	Yes	Yes	Yes

## Conclusion

This paper proposes a new KBFT algorithm for the current situation in which the PBFT algorithm and the existing new algorithm based on PBFT improvement cannot be applied to large-scale consortium chains. KBFT uses the K-prototype clustering algorithm to quickly shard the nodes in the network and uses a consensus algorithm based on the combination of the BLS multisignature and the Byzantine fault-tolerant algorithm in the shard, which markedly improves the scalability of the network and markedly reduces the communication overhead. Concurrently, through transaction sharding, the throughput increases linearly with the increase in the number of shards. The KBFT algorithm also establishes an efficient and simple credit mechanism and supervision mechanism to ensure the security and reliability of the system. For some application scenarios with high scalability requirements and high security, such as financial services, energy transactions, and the Internet of Things, the KBFT algorithm is a good solution.

However, KBFT divides all nodes in the network uniformly, but different institutions in the consortium chain are divided into layers; thus, the nodes representing institutions should also be divided into layers. Therefore, in future work, we plan to investigate how to hierarchically divide nodes.

## Data Availability

The data sets used and/or analyzed in this study are available from the corresponding authors.
